# Diagnosis of prostate cancer by multi-modal ultrasound imaging

**DOI:** 10.1097/MD.0000000000051135

**Published:** 2026-10-16

**Authors:** Jin Li, Kang He, Hao Zhang, Dejun Hu, Xin Wei, Le Luo

**Affiliations:** a Department of Ultrasound, Deyang People’s Hospital, Deyang, Sichuan, China; b Medical Records Statistics Management Section, Deyang People’s Hospital, Deyang, Sichuan, China.

**Keywords:** contrast-enhanced ultrasound, multi-modal ultrasound, prostate cancer, shear wave elastography, ultrasound-guided needle biopsy

## Abstract

To evaluate the diagnostic performance of multi-modal ultrasound imaging, combining transrectal ultrasound (TRUS), shear wave elastography (SWE), and contrast-enhanced ultrasound (CEUS), for differentiating benign and malignant prostate lesions, and to provide new evidence for the early diagnosis of prostate cancer. A total of 85 patients who prospectively underwent multi-modal ultrasound examination, ultrasound-guided prostate biopsy, and pathological and immunohistochemical marker detection at our hospital between September 2022 and June 2023 were included. All ultrasound examinations and biopsies were performed within a 2-week interval. The diagnostic performance of TRUS, SWE, CEUS, and multi-modal ultrasound imaging was compared, with 95% confidence intervals (CIs) calculated for all accuracy measures. The sensitivity, specificity, accuracy, positive predictive value, and negative predictive value were 89.80% (95% CI: 77.81 to 95.83), 86.11% (95% CI: 70.98–93.91), 88.24%, 89.80%, and 86.11% for TRUS; 85.71% (95% CI: 72.87–92.96), 72.22% (95% CI: 55.72–84.09), 80.00%, 80.77%, and 78.79% for SWE; 93.88% (95% CI: 83.13–97.97), 91.67% (95% CI: 77.46–97.20), 92.94%, 93.88%, and 91.67% for CEUS; and 97.96% (95% CI: 89.21–99.65), 94.44% (95% CI: 81.34–98.48), 96.47%, 96.00%, and 97.14% for multi-modal ultrasound imaging, respectively. The areas under the receiver operating characteristic curves (AUCs) were 0.880 (95% CI: 0.808–0.952), 0.790 (95% CI: 0.694–0.886), 0.928 (95% CI: 0.872–0.984), and 0.962 (95% CI: 0.924–1.000) for TRUS, SWE, CEUS, and multi-modal ultrasound imaging, respectively. The diagnostic performance of multi-modal ultrasound was significantly superior to that of TRUS and SWE (*P* < .05). Multi-modal ultrasound imaging demonstrates complementary diagnostic advantages, particularly for prostate tumors with atypical ultrasound features that are difficult to diagnose by a single modality. For these tumors, multi-modal ultrasound imaging improves diagnostic accuracy and provides a reliable basis for the differential diagnosis of prostate tumors, warranting further clinical application.

## 1. Introduction

Prostate cancer (PCa) is among the most prevalent malignant tumors of the male genitourinary system and has become a major health concern for men worldwide. According to GLOBOCAN 2022 estimates, PCa is the second most frequently diagnosed cancer and the fifth leading cause of cancer death among men globally.^[1,2]^ In China, the incidence of PCa has risen substantially over recent decades, and a considerable proportion of patients are already at an advanced stage at initial diagnosis.^[3]^ Early detection and timely intervention can significantly improve survival outcomes, underscoring the urgent need for effective early diagnostic strategies.^[4]^

The Chinese guideline for screening and early detection of prostate cancer (2022, Beijing) recommends serum prostate-specific antigen (PSA) testing combined with digital rectal examination as the primary screening approach for clinically suspected PCa.^[5]^ Regarding imaging, multiparametric magnetic resonance imaging (mpMRI) is recognized as the most sensitive noninvasive modality for PCa detection and localization.^[6,7]^ However, despite the adoption of the Prostate Imaging Reporting and Data System (PI-RADS v2.1), mpMRI still yields a non-negligible false-negative rate, detecting less than half of all individual PCa foci.^[8,9]^

Transrectal ultrasound (TRUS), widely used for its convenience, non-invasiveness, and affordability, has conventionally been employed for PCa screening; however, its standalone sensitivity and specificity are reported to be only approximately 50 to 60%.^[10]^ TRUS-guided prostate biopsy represents the gold standard for pathological diagnosis, yet systematic biopsy with 6 to 18 cores yields sensitivities ranging from 30 to 58% and may miss a subset of clinically significant cancers while subjecting patients to unnecessary biopsies, economic burden, and procedural complications.^[11]^

Recent advances in ultrasound technology have led to the development of shear wave elastography (SWE), which quantitatively assesses tissue stiffness, and contrast-enhanced ultrasound (CEUS), which evaluates microvascular perfusion patterns. These modalities, when combined with conventional TRUS, form a multi-modal ultrasound approach that may offer complementary diagnostic information. A recent systematic review and meta-analysis by Tang et al demonstrated that multiparametric ultrasound achieved moderate diagnostic accuracy for PCa, with a pooled sensitivity of 0.83 and specificity of 0.80, although accuracy for clinically significant PCa remained lower.^[12]^

In this study, we prospectively evaluated the diagnostic performance of multi-modal ultrasound imaging (TRUS, SWE, and CEUS) in differentiating benign from malignant prostate lesions. We further assessed whether multi-modal ultrasound imaging can serve as a reliable tool for guiding clinical decision-making in prostate cancer diagnosis.

## 2. Materials and methods

### 2.1. Study population

This prospective diagnostic accuracy study was conducted in accordance with the Standards for Reporting of Diagnostic Accuracy Studies 2015 guidelines. We prospectively enrolled consecutive patients who visited the Department of Urology at Deyang People’s Hospital between November 2022 and June 2023, met the indications for prostate biopsy, and consented to participate in this study.

Inclusion criteria were: PSA level ≥ 4 ng/mL, or abnormal digital rectal examination findings, or suspicious lesions on imaging; willingness to undergo TRUS, SWE, CEUS, and ultrasound-guided prostate biopsy; and signed informed consent. Exclusion criteria were: known allergy to ultrasound contrast agents (SonoVue); history of prostate surgery or trauma; other concurrent malignant tumors; severe cardiopulmonary disease contraindicating biopsy; and incomplete data (Fig. 1).

**Figure 1.**
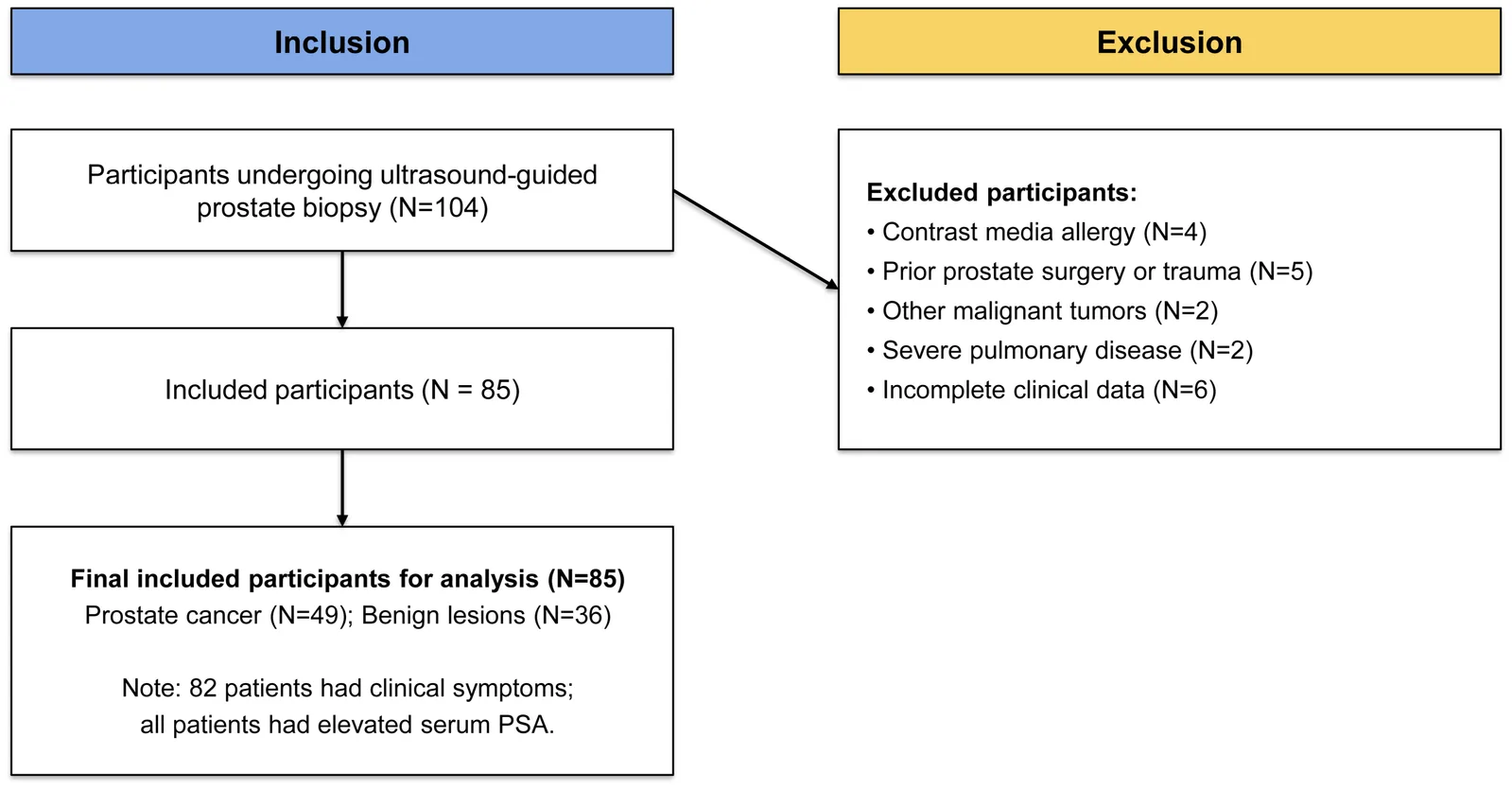
Enrollment pathway for participants. The diagram illustrates the sequential exclusion criteria applied to patients undergoing ultrasound-guided prostate biopsy, beginning with 104 individuals. After removing participants with contrast media allergy (n = 4), prior prostate surgery or trauma (n = 5), other malignant tumors (n = 2), severe pulmonary disease (n = 2), and those with incomplete data (n = 6), a final analytical sample of 85 patients was included in the study. Among them, 49 had prostate cancer and 36 had benign lesions; 82 patients presented clinical symptoms and all patients showed elevated serum prostate-specific antigen. PSA = prostate-specific antigen.

A total of 104 patients who underwent ultrasound-guided prostate biopsy were initially assessed for eligibility. After excluding 4 patients with contrast agent allergy, 5 with prior prostate surgery or trauma, 2 with other malignant tumors, 2 with severe pulmonary disease, and 6 with incomplete data, 85 patients were included in the final analysis, comprising 49 cases of prostate cancer and 36 cases of benign lesions; 82 patients presented with clinical symptoms, and serum PSA was elevated in all patients. The study flow diagram is presented in Figure 1. All patients underwent multi-modal ultrasound examination and ultrasound-guided prostate biopsy within a 2-week interval.

This study was conducted in accordance with the Declaration of Helsinki and was approved by the Ethics Committee of Deyang People’s Hospital (Ethics No.: 2022-04-085-K01; date of approval: November 25, 2022). The study was registered in the Medical Research Registration and Filing Information System of the National Healthcare Security Information Platform (Registration No.: MR-51-23-015785; date of registration: September 20, 2022). Written informed consent was obtained from all participants.

### 2.2. Multi-modal ultrasound examination

All ultrasound examinations were performed using a Mindray Resona R9T ultrasound system equipped with a dual-plane endocavitary probe (ELC 13-4U, frequency range 6–12 MHz), with real-time SWE and CEUS imaging capabilities. The examinations were conducted in the following sequence: TRUS, SWE, and CEUS. The ultrasound-to-biopsy interval was <2 weeks for all patients.

#### 2.2.1. TRUS examination

With the patient in the left lateral decubitus position, the prostate was scanned systematically from the base to the apex and from the midline to the lateral margins. The following features were assessed and recorded: bilateral symmetry of the prostate lobes, integrity of the prostate capsule, clarity of the boundary between the inner (transition zone) and outer (peripheral zone) glands, presence and echogenicity of focal lesions, margin characteristics of the lesions, and intraprostatic blood flow. Based on the ultrasound features, a region of interest (ROI) was selected, and the arterial peak systolic velocity and resistive index of the ROI were measured. The TRUS diagnostic criteria were as follows: a lesion was diagnosed as malignant when any one of the following features was present – bilateral asymmetry of the prostate lobes, an incomplete or interrupted capsule, an unclear boundary between the inner and outer glands, or a solid hypoechoic or mixed-echoic lesion with an unclear margin (Fig. 2). In addition, prostate cancer was diagnosed when color Doppler ultrasound showed grade I lesion blood flow (more than 3 punctate vessels or ≥ 1 long vessel traversing the lesion, with a length exceeding or approaching the lesion radius) or an resistive index ≥ 0.7^[10]^ (Fig. 3).

**Figure 2.**
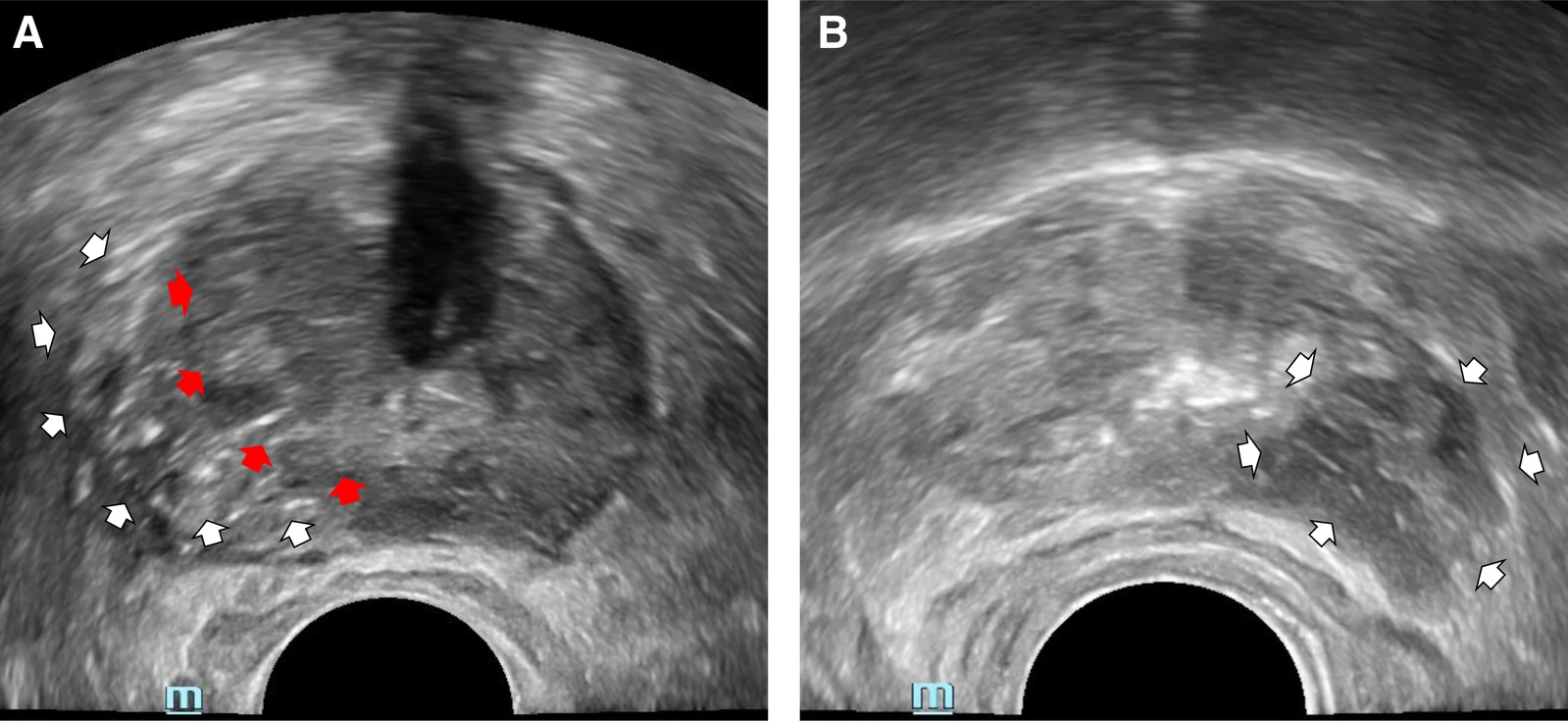
TRUS examination image of prostate cancer. (A) TRUS revealed that the bilateral prostate glands were asymmetrical, the capsule was incomplete (white arrow), and unclear boundary between inner and outer glands (red arrow); (B) TRUS showed solid hypoechoic lesions with unclear margins (white arrows). TRUS = transrectal ultrasonography.

**Figure 3.**
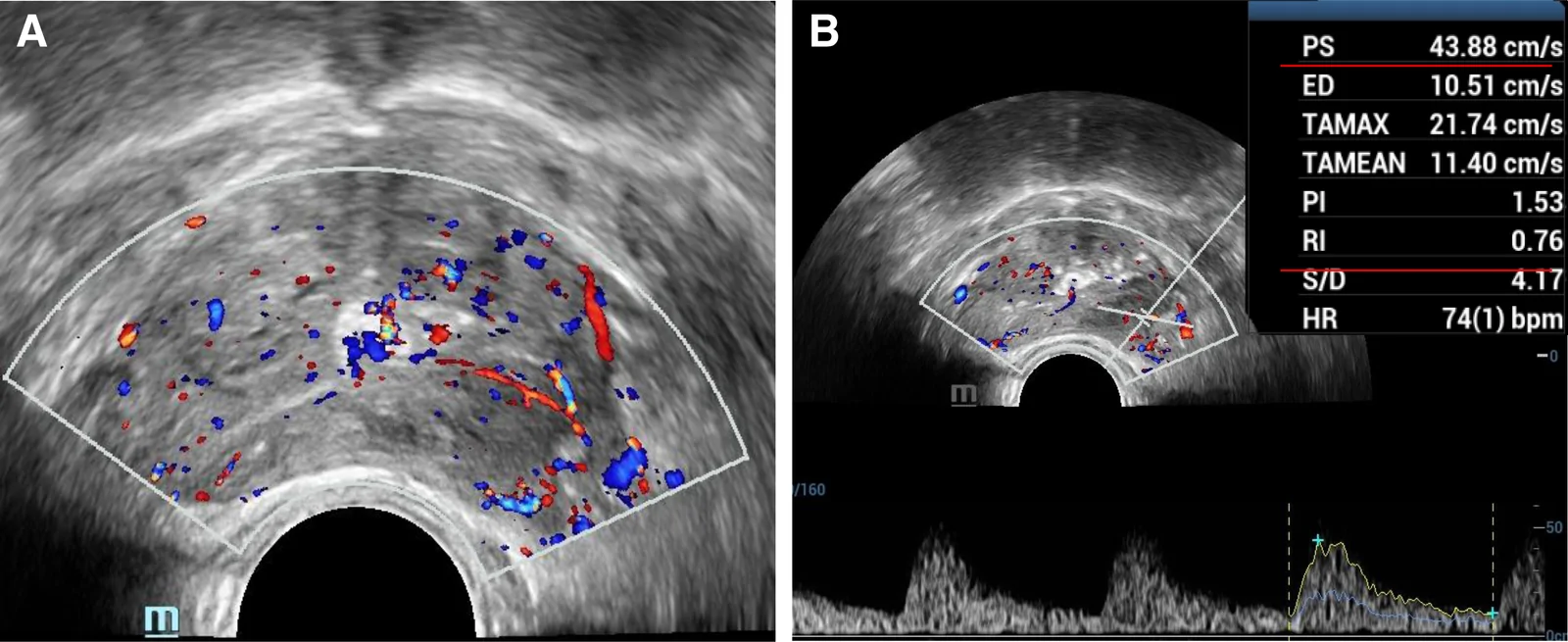
Doppler ultrasonographic images of prostate cancer. (A) The left lateral gland of the prostate was rich in blood supply with asymmetric vascular structure; (B) The peak systolic velocity (PSV) of the lesion was 43.8 cm/s, and the resistance index (RI) was 0.76.

#### 2.2.2. SWE examination

In SWE mode, a ROI was placed over the lesion or, in the absence of a focal lesion, over the peripheral zone. The maximum elastic modulus (Emax) was measured in kilopascals (kPa). After at least 3 measurements, the mean Emax value was calculated. Based on prior literature and our institutional data, an Emax cutoff value of 37 kPa was used to differentiate malignant from benign lesions, with Emax ≥ 37 kPa considered suggestive of malignancy.^[13,14]^ It should be noted that the optimal Emax threshold may vary across different populations because of differences in age, prostate volume, and ethnicity, and validation in larger cohorts is warranted. With reference to the recommendations of Supersonic Imagine (France), an Emax ≥ 37 kPa was adopted as the SWE diagnostic cutoff for prostate cancer (Fig. 4).

**Figure 4.**
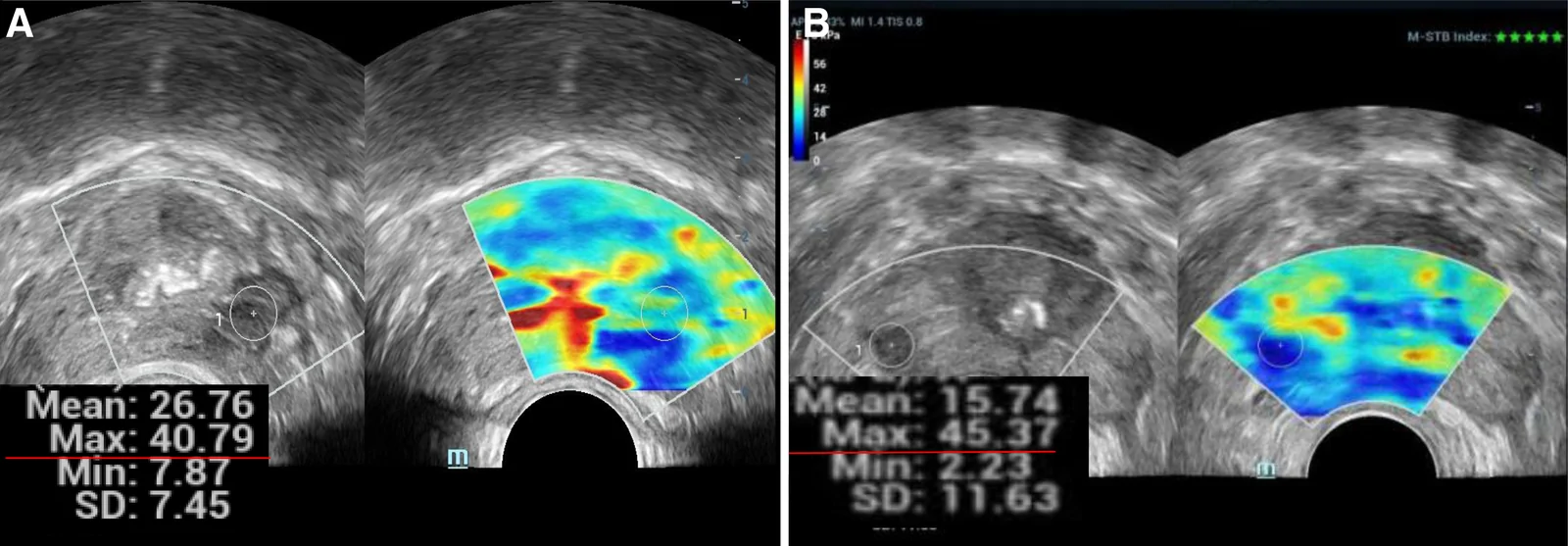
SWE of prostate cancer. (A) The maximum elastic modulus (Emax) was 40.79 kPa; (B) maximum elastic modulus (Emax) 45.37 kPa. SWE = shear wave elastography.

#### 2.2.3. CEUS examination

Contrast-enhanced ultrasound was performed after intravenous bolus injection of 2.4 mL of SonoVue (Bracco, Milan, Italy) followed by a 5 mL saline flush. The examination comprised 2 phases: arterial phase (10–45 seconds after injection): the prostate was scanned from base to apex to identify abnormal perfusion areas, particularly heterogeneous hyperperfusion; and venous phase (45–120 seconds after injection): the probe was slowly swept from apex to base to scrutinize abnormal perfusion areas, especially heterogeneous hypoperfusion. A second CEUS examination was performed after a 20-minute interval. If a ROI was identified in the first CEUS, the largest cross-section of the ROI served as the examination plane; otherwise, the maximum cross-section of the prostate was selected.

The following CEUS characteristics of the ROI were evaluated:

1. Enhancement degree: high enhancement (greater than normal prostate tissue), isoenhancement (equivalent to normal tissue), low enhancement (less than normal tissue), or no enhancement.
2. Enhancement symmetry: symmetric versus asymmetric enhancement.
3. Enhancement pattern: rapid wash-in with rapid wash-out (type I), rapid wash-in with slow wash-out (type II), synchronous enhancement and wash-out (type III), and synchronous enhancement with rapid wash-out (type IV).^[15]^

Diagnostic criteria for malignancy on CEUS included: incomplete prostate capsule after enhancement, unclear boundary between inner and outer glands, or abnormal perfusion lesions in the prostate parenchyma exhibiting rapid heterogeneous hyperenhancement, unclear boundary after enhancement, or a contrast-enhanced area larger than that on gray-scale ultrasound (Figs. 5 and 6).

**Figure 5.**
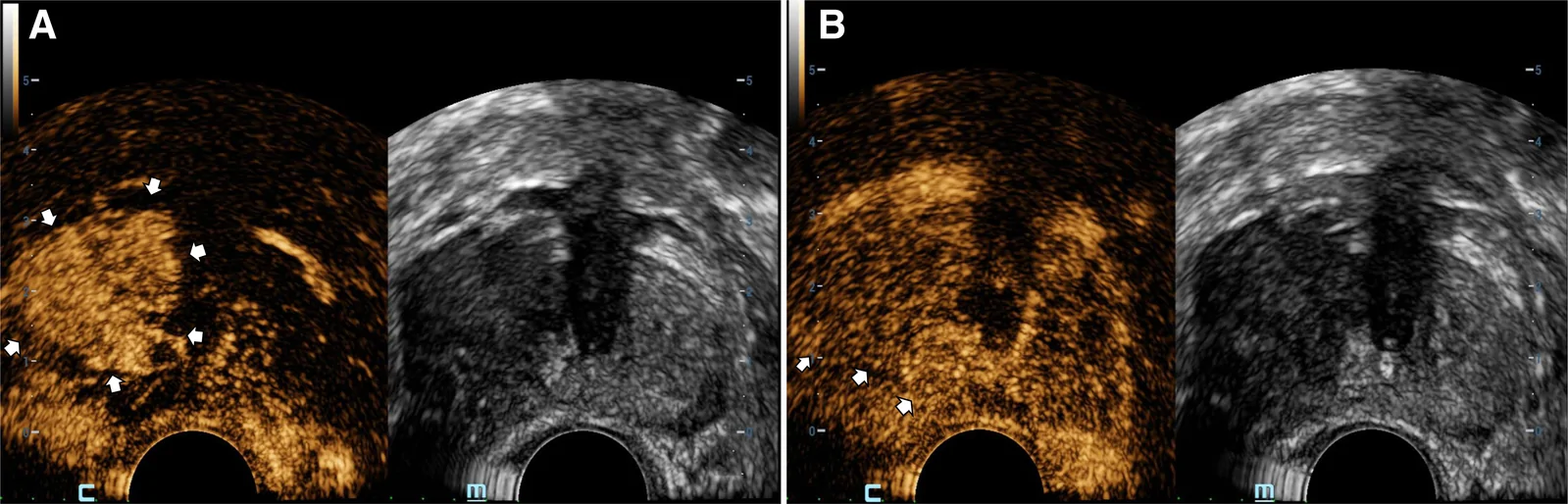
CEUS of prostate cancer. (A) During the arterial phase: the prostate showed incomplete capsule, abnormal perfusion in the parenchyma, and rapid heterogeneous hyperenhancement (white arrow); (B) During the venous phase: the lesion showed isoenhancement with unclear boundary between inner and outer glands (white arrow). CEUS = contrast-enhanced ultrasonography.

**Figure 6.**
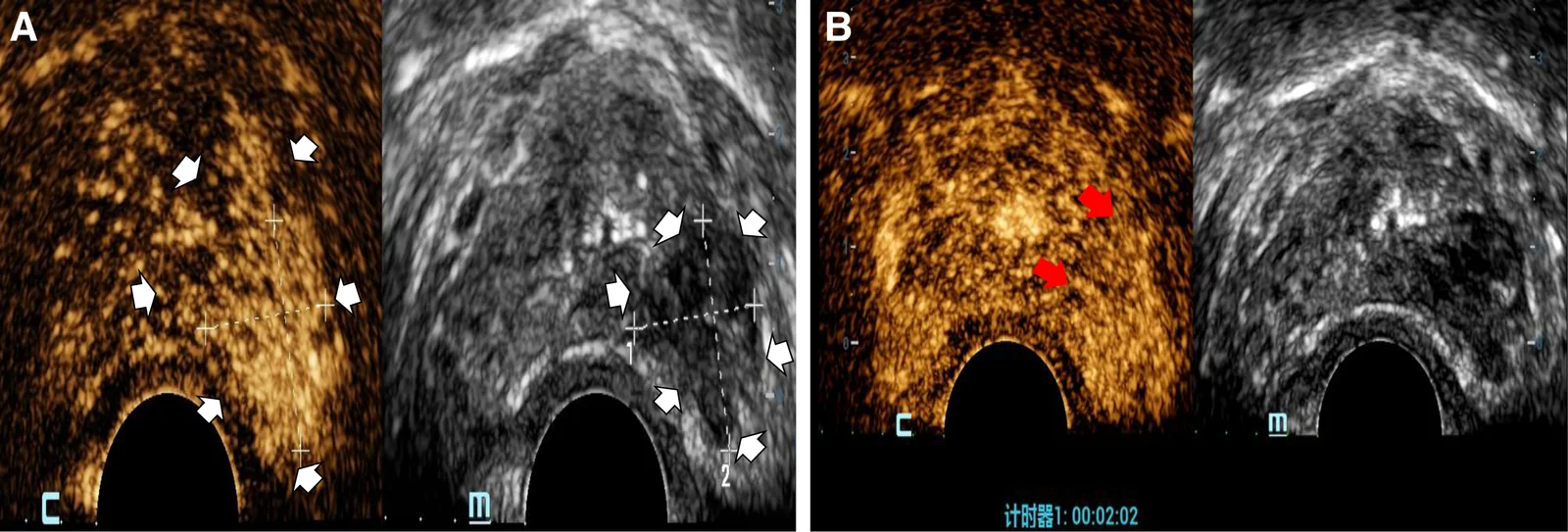
CEUS images of prostate cancer. (A) CEUS showed abnormal perfusion lesions in the prostatic parenchyma during the arterial phase. The lesions showed rapid heterogeneous hyperenhancement with unclear boundaries and a larger enhancement range than gray-scale ultrasound (white arrow). (B) CEUS showed isoenhancement of the lesion in the venous phase with unclear boundary between inner and outer glands (red arrow). CEUS = contrast-enhanced ultrasonography.

### 2.3. Ultrasound-guided prostate biopsy

All patients underwent a combination of 10-core systematic biopsy and 2 to 4-core targeted biopsy. For systematic biopsy, one core was obtained from the apex, middle, and base of each lateral prostate lobe, with 2 additional cores from the lateral peripheral zone of each lobe, and the corresponding locations were recorded. For targeted biopsy, 2 to 4 additional cores were obtained from regions of interest identified by TRUS, SWE, and CEUS, respectively.

### 2.4. Multi-modal ultrasound diagnostic criteria

The integrated multi-modal ultrasound diagnosis was defined as positive when at least 2 of the 3 modalities (TRUS, SWE, and CEUS) independently yielded a positive finding for malignancy (the “any-two-positive” rule). This criterion was adopted to balance sensitivity and specificity: requiring a single positive modality would increase sensitivity at the expense of specificity, while requiring all 3 modalities to be positive would improve specificity but substantially reduce sensitivity. The “any-two-positive” approach leverages the complementary strengths of each modality, as different ultrasound techniques capture distinct aspects of tumor biology (morphology, stiffness, and perfusion).

### 2.5. Blinding

The radiologist performing and interpreting TRUS, SWE, and CEUS examinations (J.L., with more than 5 years of experience in prostate disease diagnosis and ultrasound-guided prostate biopsy) was blinded to the pathological and immunohistochemical results. The pathologists evaluating the biopsy specimens were blinded to all ultrasound findings. All ultrasound examinations were completed and documented before the biopsy procedure, ensuring that the ultrasound interpretations were not influenced by pathological outcomes.

### 2.6. Statistical methods

Statistical analyses were performed using SPSS 27.0 (IBM Corp., Armonk). Continuous variables were expressed as mean ± standard deviation and compared using the independent samples *t* test. Categorical variables were expressed as frequencies and percentages and compared using the Pearson chi-square test or Fisher exact test, as appropriate. The diagnostic performance of each modality was assessed by calculating sensitivity, specificity, accuracy, positive predictive value (PPV), and negative predictive value (NPV), with 95% confidence intervals (CIs) estimated using the Wilson score method. Receiver operating characteristic curves were constructed, and the area under the curve (AUC) with 95% CIs was computed. AUC comparisons were performed using the DeLong test. A two-sided *P* value < .05 was considered statistically significant.

## 3. Results

### 3.1. Patient characteristics

The clinical and demographic characteristics of the 85 enrolled patients are summarized in Table 1. The mean age was 73.56 ± 6.92 years for the benign group (n = 36) and 72.92 ± 8.29 years for the malignant group (n = 49), with no statistically significant difference (*P* = .71). PSA levels were significantly higher in the malignant group (63.82 ± 39.2 ng/mL) than in the benign group (17.7 ± 16.7 ng/mL, *P* < .001). Prostate volume was significantly smaller in the malignant group (43.05 ± 20.3 cm^3^) compared with the benign group (54.08 ± 21.7 cm^3^, *P* = .019). PSA density was significantly higher in the malignant group (1.70 ± 1.32 ng/mL·cm^3^) compared with the benign group (0.35 ± 0.29 ng/mL·cm^3^, *P* < .001).

**Table 1:** Comparison of general information between benign and malignant prostate lesions.

Pathological diagnosis	Number (n)	Average age (yr)	PSA (ng/mL)	Prostate volume (cm^3^)	PSA density (ng/mL·cm^3^)
Benign	36	73.56 ± 6.92	17.7 ± 16.7	54.08 ± 21.7	0.35 ± 0.29
Malignant	49	72.92 ± 8.29	63.82 ± 39.2	43.05 ± 20.3	1.70 ± 1.32
*t*		0.37	−6.6	2.39	−6.032
*P*		.71	<.000	.019	<.000

PSA = prostatic-specific antigen, PSA density = PSA/prostate volume.

### 3.2. TRUS results

Among the 85 patients, TRUS imaging demonstrated the following characteristics: bilateral asymmetry in 35 cases (41.18%), incomplete or interrupted capsule in 37 cases (43.53%), unclear boundary between inner and outer glands in 38 cases (44.71%), and visible focal lesions in 72 cases (84.71%). Hypoechoic or mixed-echoic lesions were observed in 51 cases (60.00%). Based on TRUS findings, 36 tumors were classified as benign and 49 as malignant. Comparison with pathological diagnosis revealed that 6 malignant tumors were misdiagnosed as benign (false negatives) and 5 benign tumors were misdiagnosed as malignant (false positives). The detailed cross-tabulation is presented in Table 2.

**Table 2:** TRUS, SWE, CEUS, multi-model ultrasound identification benign and malignant prostate tumors and pathological diagnosis results.

Pathological diagnosis	Number (n)	TRUS		SWE		CEUS		Multi-model ultrasound	
		Malignant (n)	Benign (n)	Malignant (n)	Benign (n)	Malignant (n)	Benign (n)	Malignant (n)	Benign (n)
Malignant	49	44	5	42	10	46	3	48	2
Benign	36	5	31	7	26	3	33	1	34

CEUS = contrast-enhanced ultrasonography, SWE = shear wave elastography, TRUS = transrectal ultrasonography.

### 3.3. SWE results

Of the 85 patients, 57 had an Emax value ≥ 37 kPa and 28 had an Emax < 37 kPa. SWE-based diagnosis misclassified 7 malignant tumors with Emax below the cutoff as benign (false negatives) and 10 benign tumors with Emax above the cutoff as malignant (false positives) (Table 2). The sensitivity of SWE (85.71%) was higher than that of TRUS, but its specificity (72.22%) was lower.

### 3.4. CEUS results

On CEUS imaging, 58 of the 85 patients (68.24%) demonstrated visible lesions. The CEUS characteristics were as follows: incomplete prostate capsule in 37 cases (43.53%), unclear boundary between inner and outer glands in 41 cases (48.24%), and abnormal perfusion lesions in 58 cases (68.24%). With respect to enhancement degree, 45 cases (77.59%) showed high enhancement, 9 cases (15.52%) showed isoenhancement, and 4 cases (6.90%) showed low enhancement. Regarding enhancement pattern, 10 cases (17.24%) exhibited rapid wash-in with rapid wash-out (type I), 38 cases (65.52%) exhibited rapid wash-in with slow wash-out (type II), 6 cases (10.34%) showed synchronous enhancement and wash-out (type III), and 4 cases (6.90%) showed synchronous enhancement with rapid wash-out (type IV). CEUS misdiagnosed 3 malignant tumors as benign and 3 benign tumors as malignant (Table 2). Notably, rapid wash-out (type I) was observed in only 10 of the 47 malignant lesions with abnormal perfusion (21.28%), and no significant difference in rapid contrast wash-out was observed between the benign and malignant groups.

### 3.5. Multi-modal ultrasound results

Using the integrated “any-two-positive” multi-modal diagnostic criterion, 50 patients were diagnosed as malignant (Fig. 7) and 35 as benign (Fig. 8). Compared with pathological diagnosis, the multi-modal approach misdiagnosed only 2 malignant tumors as benign and 1 benign tumor as malignant (Table 2), representing the lowest misdiagnosis rate among all modalities.

**Figure 7.**
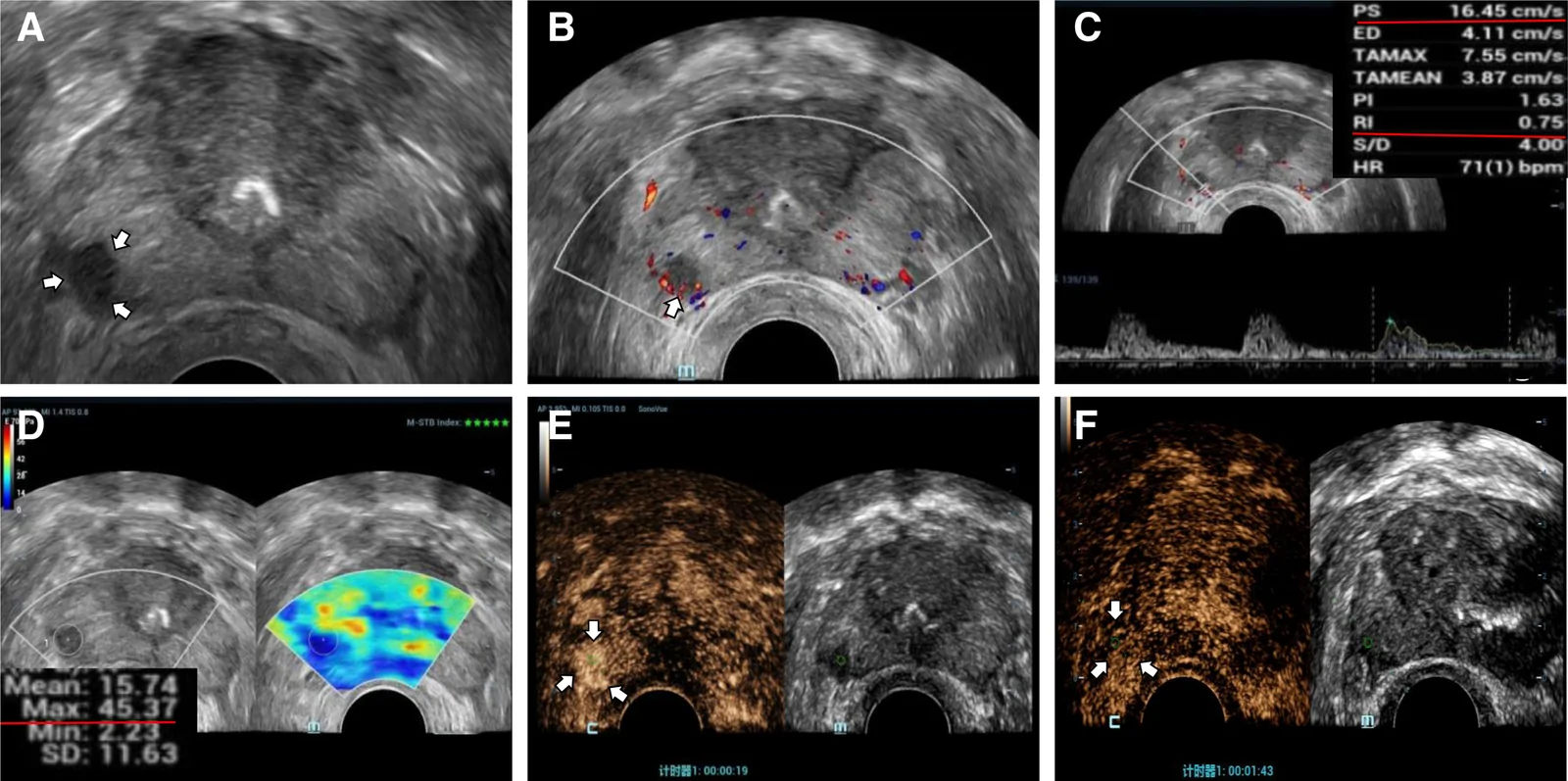
Multimodal ultrasound images of prostate cancer. (A) TRUS showed a hypoechoic lesion in the right peripheral gland region (white arrow); (B) rich blood flow signals detected by color Doppler (white arrow); (C) The peak systolic velocity (PSV) was 16.46 cm/s, and the resistance index (RI) was 0.75. (D) The maximum elastic modulus of SWE was 45.37 kPa. (E) CEUS showed rapid heterogeneous hyperenhancement with unclear boundary in the early stage (white arrow). (F) CEUS showed isoenhancement in the venous phase of the lesion (white arrow). CEUS = contrast-enhanced ultrasonography, SWE = shear wave elastography, TRUS = transrectal ultrasonography.

**Figure 8.**
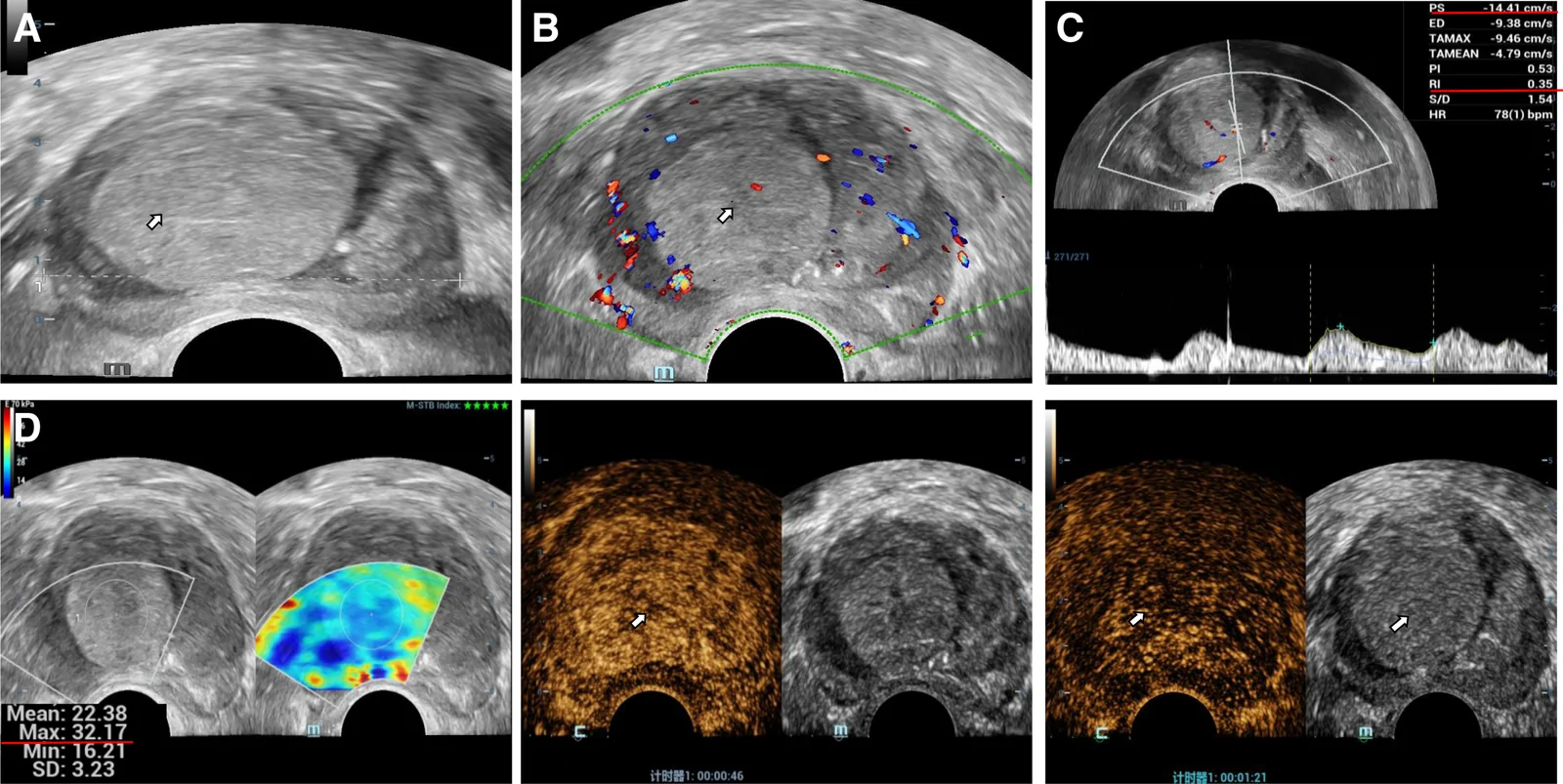
Multimodal ultrasound images of prostatic hyperplasia. (A) TRUS showed isoechoic lesions in the right internal gland region (white arrow); (B) a few blood flow signals detected by color Doppler (white arrow); (C) Peak blood flow velocity (PSV) was 14.41 cm/s and resistance index (RI) was 0.35. (D) The maximum elastic modulus was 32.17 kPa. (E) CEUS showed heterogeneous isoenhancement in the arterial phase (white arrow). (F) CEUS showed isoenhancement in the venous phase of the lesion (white arrow). CEUS = contrast-enhanced ultrasonography, TRUS = transrectal ultrasonography.

### 3.6. Comparison of diagnostic performance

The diagnostic performance metrics for all 4 modalities are presented in Table 3. The multi-modal ultrasound approach achieved the highest sensitivity (97.96%, 95% CI: 89.21–99.65), specificity (94.44%, 95% CI: 81.34–98.48), accuracy (96.47%), PPV (96.00%), and NPV (97.14%). The AUC values were 0.880 (95% CI: 0.808–0.952), 0.790 (95% CI: 0.694–0.886), 0.928 (95% CI: 0.872–0.984), and 0.962 (95% CI: 0.924–1.000) for TRUS, SWE, CEUS, and multi-modal ultrasound, respectively (Fig. 9). In pairwise AUC comparisons using the DeLong test (Table 4), the multi-modal approach was significantly superior to TRUS (*Z* = 2.434, *P* = .015) and SWE (*Z* = 3.388, *P* = .001), but not significantly different from CEUS (*Z* = 1.722, *P* = .085). CEUS was significantly superior to SWE (*Z* = 2.469, *P* = .014) but not to TRUS (*Z* = 1.200, *P* = .230).

**Table 3:** Diagnostic performance of TRUS, CEUS, SWE, multi-model ultrasound in 85 patients.

Methods	Area	Sensitivity (%)	Specificity (%)	Accuracy (%)	PPV (%)	NPV (%)
TRUS	0.880 (0.808–0.952)	89.80 (77.81–95.83)	86.11 (70.98–93.91)	88.23	89.80	86.11
SWE	0.790 (0.694–0.886)	85.71 (72.87–92.96)	72.22 (55.72–84.09)	80.00	80.77	78.79
CEUS	0.928 (0.872–0.984)	93.88 (83.13–97.97)	91.67 (77.46–97.20)	92.94	93.88	91.67
Multi-model	0.962 (0.924–1.000)	97.96 (89.21–99.65)	94.44 (81.34–98.48)	96.47	96.00	97.14

CEUS = contrast-enhanced ultrasonography, NPV = negative predictive value, PPV = positive predictive value, SWE = shear wave elastography, TRUS = transrectal ultrasonography.

**Table 4:** Comparison of TRUS, SWE, CEUS and multi-mode ultrasound in paired-sample area difference under the ROC curves.

Test result pair(s)	Asymptotic			AUC difference	Asymptotic 95% confidence interval
	*Z*	*P*			
Multi-model - CEUS	1.722		.085	0.034	−0.005–0.073
Multi-model - SWE	3.388		.001	0.172	0.073–0.272
Multi-model - TRUS	2.434		.015	0.082	0.016–0.149
CEUS - SWE	2.469		.014	0.138	0.028–0.248
CEUS - TRUS	1.2		.23	0.048	−0.031–0.127
TRUS - SWE	1.402		.161	0.09	−0.215–0.036

AUC = area under the ROC curve, CEUS = contrast-enhanced ultrasonography, ROC = receiver operating characteristic, SWE = shear wave elastography, TRUS = transrectal ultrasonography.

**Figure 9.**
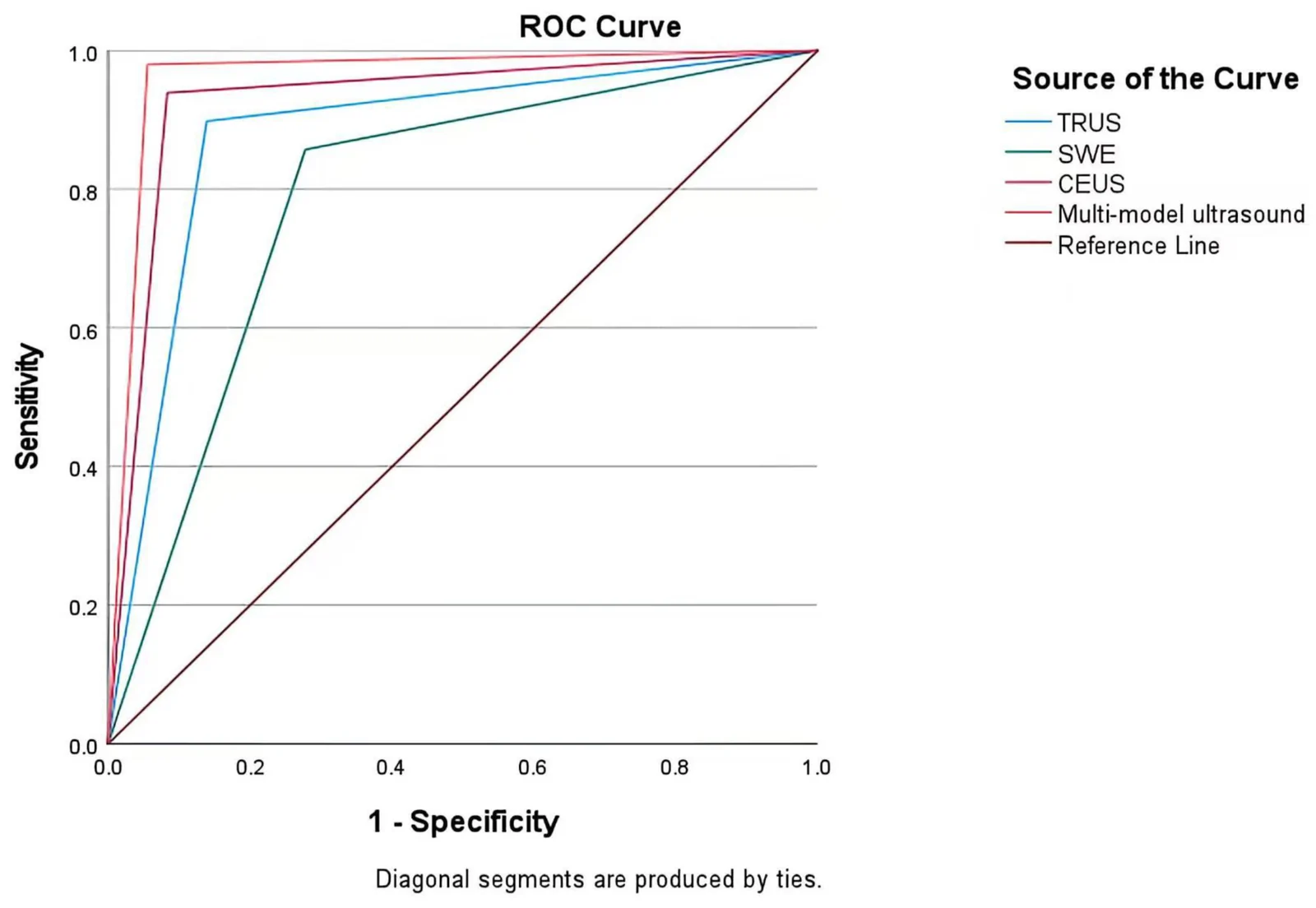
ROC curves of TRUS, SWE, and CEUS in evaluating benign and malignant prostate lesions. CEUS = contrast-enhanced ultrasonography, ROC = receiver operating characteristic, SWE = shear wave elastography, TRUS =transrectal ultrasonography.

## 4. Discussion

In this prospective diagnostic accuracy study of 85 patients with suspected prostate cancer, we demonstrated that multi-modal ultrasound imaging combining TRUS, SWE, and CEUS yielded excellent diagnostic performance, with an AUC of 0.962, sensitivity of 97.96%, and specificity of 94.44%. The multi-modal approach significantly outperformed individual modalities, particularly TRUS and SWE, affirming the complementary nature of these ultrasound techniques.

TRUS remains the most widely used ultrasound modality for prostate evaluation. In our study, TRUS achieved an accuracy of 88.24% based on morphological features including lobe asymmetry, capsular integrity, glandular boundary clarity, and lesion echogenicity. These findings are consistent with previous reports showing that malignant prostate lesions commonly present with asymmetric lobes, capsular interruption, and hypoechoic or mixed-echoic solid lesions.^[16]^ However, TRUS is limited in detecting isoechoic or small lesions that do not produce appreciable mass effect. Doppler ultrasound can aid in detecting focal increases in blood flow signal; however, inflammatory lesions may also produce elevated Doppler signals, reducing specificity.^[17]^

SWE quantifies tissue stiffness by measuring the shear wave elastic modulus, providing a more objective assessment of tissue hardness. In our cohort, using an Emax cutoff of 37 kPa, SWE achieved a sensitivity of 85.71% and accuracy of 80.00%. The higher sensitivity of SWE relative to TRUS may be attributable to its ability to detect small or infiltrative malignant lesions that are not apparent on gray-scale ultrasound due to increased tissue stiffness. Conversely, the lower specificity may be explained by reduced elastographic accuracy in the setting of large prostate volumes or deep-seated lesions, which are common in elderly patients with benign prostatic hyperplasia.^[18,19]^ Importantly, the optimal Emax cutoff value is population-dependent and may require adjustment in different clinical settings based on local prevalence, age distribution, and prostate volume characteristics.

CEUS leverages the nonlinear acoustic properties of microbubble contrast agents to depict tissue perfusion and hemodynamics.^[20]^ In the present study, CEUS demonstrated the highest standalone performance among the 3 modalities (AUC 0.928), with characteristic features including incomplete capsule, unclear inner-outer gland boundary, and abnormal perfusion patterns. This study found that not all prostate malignant lesions showed incomplete capsule, unclear boundary between inner and outer glands after enhanced ultrasound, and abnormal perfusion of contrast medium in the prostate parenchyma, but the risk of prostate malignant lesions with the above characteristics was significantly increased. The reason may be due to the erosion and destruction of normal prostate tissue by advanced prostate malignant lesions and the invasion of prostate capsule and surrounding tissues, which is consistent with the multi-center research data in China showing that most patients with prostate cancer in China are in the middle and advanced stages when they are initially diagnosed.^[21]^ We observed that the majority of malignant lesions exhibited rapid wash-in with slow wash-out (type II, 65.52%), rather than the classic rapid wash-in and rapid wash-out (type I, only 17.24%) pattern frequently cited in earlier studies. This discrepancy may reflect differences in tumor biology, stage distribution, or technical parameters across studies and warrants further investigation.

The integrated multi-modal approach using the “any-two-positive” criterion achieved the best diagnostic performance, highlighting the advantage of combining morphological (TRUS), mechanical (SWE), and functional (CEUS) information. The complementary nature of these modalities is particularly valuable for atypical lesions that may be missed by any single technique. Our findings align with the meta-analysis by Tang et al,^[12]^ which reported that multiparametric ultrasound provides moderate-to-high diagnostic accuracy for PCa, and extend these observations by demonstrating the incremental benefit of the specific three-modality combination employed in this study.

This study had several limitations that should be acknowledged. First, the sample size (n = 85) was relatively modest and derived from a single center, which may limit the generalizability of the findings. Second, although we employed blinding of the ultrasound operator to pathological results and of the pathologists to ultrasound findings, the inherent operator-dependence of ultrasound techniques cannot be fully eliminated. Third, the optimal Emax cutoff of 37 kPa was derived from a combination of prior literature and local data; external validation in independent cohorts is necessary. Fourth, we did not perform a formal sample size calculation, and the 95% CIs for some estimates, particularly specificity, were relatively wide. Fifth, the lack of mpMRI as a comparator limits our ability to position multi-modal ultrasound within the broader diagnostic pathway. Future multicenter studies with larger sample sizes are warranted to validate these findings and to establish the role of multi-modal ultrasound in routine clinical practice.

## 5. Conclusion

Multi-modal ultrasound imaging combining TRUS, SWE, and CEUS demonstrates complementary diagnostic advantages and achieves high diagnostic accuracy for differentiating benign and malignant prostate lesions. This approach is particularly valuable for tumors with atypical ultrasound features that are challenging to diagnose by a single modality alone. Multi-modal ultrasound imaging can serve as a reliable basis for the differential diagnosis of prostate tumors and holds promise for guiding clinical decision-making in prostate cancer management.

## Author contributions

**Conceptualization:** Kang He.

**Data curation:** Jin Li, Le Luo, Hao Zhang, Dejun Hu, Xin Wei.

**Formal analysis:** Jin Li, Le Luo, Hao Zhang, Xin Wei.

**Investigation:** Le Luo, Kang He.

**Methodology:** Kang He.

**Resources:** Jin Li.

**Writing—original draft:** Jin Li, Hao Zhang, Dejun Hu.

**Writing—review & editing:** Jin Li, Le Luo, Xin Wei.

## References

[1] Sung H Ferlay J Siegel RL. Global cancer statistics 2020: GLOBOCAN estimates of incidence and mortality worldwide for 36 cancers in 185 countries. CA Cancer J Clin. 2021;71:209–49. 10.3322/caac.21660 33538338

[2] Bray F Laversanne M Sung H. Global cancer statistics 2022: GLOBOCAN estimates of incidence and mortality worldwide for 36 cancers in 185 countries. CA Cancer J Clin. 2024;74:229–63. 10.3322/caac.21834 38572751

[3] Wang Z Zhu S Tan S Zeng Y Zeng H. The P2 purinoceptors in prostate cancer. Purinergic Signal. 2023;19:255–63. 10.1007/s11302-022-09874-2 PMC9984634 35771310

[4] Hartley A Ahmad I. The role of PPARγ in prostate cancer development and progression. Br J Cancer. 2023;128:940–5. 10.1038/s41416-022-02096-8 PMC10006070 36510001

[5] He J Chen WQ Li N.; China Prostate Cancer Screening and Early Diagnosis and Early Treatment Guidelines Development Expert Group. China guideline for the screening and early detection of prostate cancer (2022, Beijing). Zhonghua Zhong Liu Za Zhi. 2022;44:29–53. 10.3760/cma.j.cn112152-20211226-00975 35073647

[6] Liu W Patil D Howard DH. Adoption of prebiopsy magnetic resonance imaging for men undergoing prostate biopsy in the United States. Urology. 2018;117:57–63. 10.1016/j.urology.2018.04.007 29679601

[7] Turkbey B Rosenkrantz AB Haider MA. Prostate imaging reporting and data system version 2.1: 2019 update of prostate imaging reporting and data system version 2. Eur Urol. 2019;76:340–51. 10.1016/j.eururo.2019.02.033 30898406

[8] Johnson DC Raman SS Mirak SA. Detection of individual prostate cancer foci via multiparametric magnetic resonance imaging. Eur Urol. 2019;75:712–20. 10.1016/j.eururo.2018.11.031 30509763

[9] Vassallo R Mannas MP Salcudean SE. Developments in ultrasound-based imaging for prostate cancer detection. Prostate. 2025;85:823–32. 10.1002/pros.24893 PMC12068032 40152157

[10] Liu Y Zeng S Xu R. Application of multiple ultrasonic techniques in the diagnosis of prostate cancer. Front Oncol. 2022;12:905087. 10.3389/fonc.2022.905087 PMC9271763 35832558

[11] Grabski B Baeurle L Loch A. Computerized transrectal ultrasound of the prostate in a multicenter setup (C-TRUS-MS): detection of cancer after multiple negative systematic random and in primary biopsies. World J Urol. 2011;29:573–9. 10.1007/s00345-011-0713-0 21691722

[12] Tang Y Li X Jiang Q Zhai L. Diagnostic accuracy of multiparametric ultrasound in the diagnosis of prostate cancer: systematic review and meta-analysis. Insights Imaging. 2023;14:203. 10.1186/s13244-023-01543-1 PMC10673798 38001351

[13] Barr RG Memo R Schaub CR. Shear wave ultrasound elastography of the prostate: initial results. Ultrasound Q. 2012;28:13–20. 10.1097/RUQ.0b013e318249f594 22357224

[14] Barr RG Cosgrove D Brock M. WFUMB guidelines and recommendations on the clinical use of ultrasound elastography: part 5. Prostate. Ultrasound Med Biol. 2017;43:27–48. 10.1016/j.ultrasmedbio.2016.06.020 27567060

[15] Liu Y Xiang L Xu G Zhang Y Xu H. Recent advances of multimodal ultrasound in image-guided prostate-targeted biopsy. J Interv Med. 2022;5:117–21. 10.1016/j.jimed.2022.04.005 PMC9617149 36317149

[16] Zhang X Hong H Liang D. The combined value of mpUS and mpMRI-TRUS fusion for the diagnosis of clinically significant prostate cancer. Cancer Imaging. 2022;22:60. 10.1186/s40644-022-00498-8 PMC9580162 36258247

[17] Tang J Li S Li J. Correlation between prostate cancer grade and vascularity on color Doppler imaging: preliminary findings. J Clin Ultrasound. 2003;31:61–8. 10.1002/jcu.10139 12539246

[18] Ageeli W Wei C Zhang X. Quantitative ultrasound shear wave elastography (USWE)-measured tissue stiffness correlates with PIRADS scoring of MRI and Gleason score on whole-mount histopathology of prostate cancer: implications for ultrasound image-guided targeting approach. Insights Imaging. 2021;12:96. 10.1186/s13244-021-01039-w PMC8266979 34236553

[19] Woo S Suh CH Kim SY Cho JY Kim SH. Shear-wave elastography for detection of prostate cancer: a systematic review and diagnostic meta-analysis. AJR Am J Roentgenol. 2017;209:806–14. 10.2214/AJR.17.18056 28796546

[20] Sharen GW Zhang J. Application of shear wave elastography and contrast-enhanced ultrasound in transrectal prostate biopsy. Curr Med Sci. 2022;42:447–52. 10.1007/s11596-022-2484-1 35301673

[21] Lu DY Liu L Shen L Cai JR Xu L Xiang LH. Comparison of contrast-enhanced ultrasound targeted biopsies versus standard systematic biopsies for prostate cancer correction in different PSA value groups in rural China. Clin Hemorheol Microcirc. 2021;77:295–301. 10.3233/CH-200993 33252065

